# Prevalence of gingivitis and risk factors among pregnant women from Acapulco, Guerrero: a cross-sectional study

**DOI:** 10.21142/2523-2754-1001-2022-094

**Published:** 2022-03-30

**Authors:** Lisa Madison Gallardo Chávez, Jordy Mizrraim Rodríguez Díaz, Carlos Alberto Juárez Medel, Janitzie Hernández Clemente, Arnold Uriel Herrera Santos

**Affiliations:** 1 Clinica Medica San Jose, Acapulco. Guerrero, Mexico. leslie213004@hotmail.com, jmrd-88@hotmail.com Clinica Medica San Jose Acapulco. Guerrero Mexico leslie213004@hotmail.com jmrd-88@hotmail.com; 2 Departamento de Apoyo a Investigacion de la Coordinacion de Investigacion Educativa. Coordinacion de Formacion y Capacitacion del Personal de Salud de la Unidad de Coordinacion Nacional Medica del Instituto de Salud para el Bienestar, Acapulco. Guerrero, Mexico. dr.charly.jume@hotmail.com Departamento de Apoyo a Investigacion de la Coordinacion de Investigacion Educativa Coordinacion de Formacion y Capacitacion del Personal de Salud de la Unidad de Coordinacion Nacional Medica Instituto de Salud para el Bienestar Acapulco. Guerrero Mexico dr.charly.jume@hotmail.com; 3 Jurisdiccion Sanitaria de la Secretaria de Salud, Acapulco. Guerrero, Mexico. drajanitzie@hotmail.com Jurisdiccion Sanitaria de la Secretaria de Salud Acapulco. Guerrero Mexico drajanitzie@hotmail.com; 4 Division de Periodoncia, Universidad Tecnologica de Mexico. Ciudad de Mexico, Mexico. arnold_hs@outlook.com Universidad Tecnologica de Mexico Division de Periodoncia Universidad Tecnologica de Mexico Ciudad de Mexico Mexico arnold_hs@outlook.com

**Keywords:** pregnancy, gingivitis, pregnant women, embarazo, gingivitis, mujeres embarazadas

## Abstract

**Introduction:**

Gingivitis is a condition that has been associated with an exaggerated inflammatory response of the gums to oral biofilm, attributed to the secretion of hormones during pregnancy.

**Objectives:**

To estimate the prevalence of gingivitis and identify associated risk factors among pregnant women in a private medical clinic from Acapulco, Guerrero.

**Material and methods:**

Cross-sectional study in a convenience sample of 92 pregnant women, during the period from January to March 2020. A self-administered questionnaire collected sociodemographic data, economic income, oral hygiene habits and personal pathological history. The periodontium was evaluated based on the new classification of gingival health and gingivitis induced by oral biofilm on six representative teeth. Multivariate analysis identified factors associated with gingivitis using the odds ratio and its 95% confidence interval as an estimate of the strength of association with CIETmap statistical software.

**Results:**

The prevalence of gingivitis was 60% (55/92). Two factors remained in the final model of the multivariate analysis. The main associated strength was found in the variable of not using oral hygiene aids (ORa= 6.76; CI 95%= 2.01-22.78). The other variable was not attending dental visits (ORa= 3.74; CI 95%= 1.44-9.73).

**Conclusion:**

Gingivitis affected about six out of ten pregnant women. Knowing the risk factors, it will be important to reinforce health education strategies and the importance of clinical monitoring during pregnancy.

## RESUMEN

**Introducción:**

La gingivitis es una afección que se asocia a una respuesta inflamatoria exagerada de las encías al biofilm oral, por la secreación de hormonas durante el embarazo.

**Objetivos:** Estimar la prevalencia de gingivitis e identificar factores de riesgo asociados en gestantes de una clínica médica privada de Acapulco, Guerrero.

**Materiales y métodos:**

Estudio transversal en una muestra por conveniencia de 92 gestantes, durante el periodo de enero a marzo del 2020. Un cuestionario autoadministrado recolectó datos sociodemográficos, ingreso económico, hábitos de higiene oral y antecedentes personales patológicos. Se evaluó el periodonto con base en la nueva clasificación de salud gingival y gingivitis inducida por biofilm sobre seis dientes representativos. El análisis multivariado identificó factores asociados a gingivitis utilizando la razón de momios y su intervalo de confianza del 95% como estimación de la fuerza de asociación con el *software* estadístico CIETmap.

**Resultados:**

La prevalencia de gingivitis fue del 60% (55/92). Dos factores se mantuvieron en el modelo final del análisis multivariado. La mayor fuerza de asociación fue en la variable de no usar auxiliares de higiene oral (ORa = 6.76; IC 95% = 2.01-22.78). La otra variable fue el no acudir a visita odontológica (ORa = 3.74; IC 95% = 1.44-9.73).

**Conclusión:**

La gingivitis afecta a seis de cada diez mujeres embarazadas. Al ser conocidos los factores de riesgo, será importante reforzar las estrategias educativas sobre el ciudado de la salud y la importancia del control clínico durante el embarazo.

## INTRODUCTION

During pregnancy, a woman's body undergoes many alterations for the development of the fetus, where changes in hormone production occur.^1^^-^^3^ These physiological conditions have an influence on the oral health of pregnant women.^4^^,^^5^ It is estimated that one in ten women suffer from some oral mucosal disorder during pregnancy.^5^ Gingivitis is one of the most common oral cavity disorders during pregnancy. ^(^^3^^,^^6^ It is associated with an exaggerated inflammatory response of the gums to oral biofilm, attributed to the secretion of hormones. ^(^^2^^,^^3^

The prevalence of gingivitis in pregnant women varies in different parts of the world; in Asia, at the hospital level, it is estimated that the average frequency is 55%.^7^^-^^9^ In Africa it is reported to affect seven out of every ten pregnant women attended at the tertiary hospital level. ^(^^10^^,^^11^ In Latin America it affects three quarters of pregnant women attended in hospitals. ^(^^12^^-^^16^ A study carried out in a family medicine unit of the Mexican Social Security Institute in the city of Acapulco, found that the prevalence of biofilm and periodontal disease in pregnant women was 74% and 65%, respectively. ^(^^17^

It has been found that the etiology of periodontal disease during pregnancy is multicausal. Some studies mention that they are due to hormonal changes,^1^^-^^3^ poor oral hygiene,^5^^,^^13^^,^^14^^,^^17^ level of schooling,^5^^,^^12^ belonging to an ethnic group,^5^^,^^13^ maternal age,^7^^,^^9^^,^^10^ socioeconomic factors,^7^^,^^12^ periodontal bleeding,^9^ and not attending a dental consultation.^16^^,^^18^ Hormonal changes influence the reproduction of microorganisms associated with periodontopathies, such as *Bacteroides intermedius* during the second trimester; *Porphyromonas gingivalis* and *Tannerella forsythia* in the 12th week of gestation; *Prevotella intermedia*, *Fusobacterium nucleatum* and *Aggregatibacter actinomycetemcomitans* that have been found in the saliva of pregnant women.^3^

Periodontal disease has been documented to be associated with low birth weight, preterm delivery and preeclampsia. Cytokines and other inflammatory mediators generated in the immune response to the infection would disseminate through the bloodstream until they reach the uterine cavity, where they promote the synthesis of prostaglandins, involved in the natural history of preterm labor.^6^ Therefore, it is important to address intervention strategies to control oral inflammatory disease in order to decrease the systemic inflammatory burden and ultimately reduce the potential for adverse pregnancy outcomes. Intervention on oral health promotion in pregnant women becomes a strategy that helps to reinforce knowledge about oral health care during this period.^19^^-^^21^

The interest in gingival alterations is based not so much on their severity, but on their enormous prevalence in pregnant women,^9^^,^^11^^,^^14^^,^^15^ being more frequent in the second,^10^ and third trimester of gestation.^8^^,^^15^ Given the results of studies that have found that gestation exacerbates the gingival response to oral biofilm and that relate it to periodontal disease, there is a need for health education in pregnant women.^10^^,^^18^^,^^21^ In our region, only one descriptive study has reported the situation of the event, but it was in a public sector hospital. There are no records on the epidemiology of gingivitis and associated factors at the private level.

The objective of this study was to estimate the prevalence of gingivitis and identify associated factors among pregnant women in a private medical clinic from Acapulco, Guerrero, Mexico.

## MATERIAL AND METHODS

Cross-sectional study in pregnant women at San Jose medical clinic from Acapulco, Guerrero, Mexico; during the period from January to March 2020. For ease, a convenience sample of 103 pregnant women was obtained; however, four were excluded because of the presence of orthodontic appliances, two with crowding and two with a history of periodontal treatment. Three were eliminated because they refused to participate, leaving a total of 92 pregnant women who attended a general dental consultation. 

Prior to the fieldwork, four dental interns were standardized in order to collect the information and perform the clinical oral examination. The calibration of the examiners began with theoretical training, continued with clinical practice in a health center and was validated in the pilot test, with the diagnostic concordance of a specialist in periodontics, which was a good overall parameter (Fleiss' Kappa=0.71).^22^ A self-administered questionnaire of 14 items was applied, which collected sociodemographic information (age, locality, degree of studies and marital status), monthly economic income, pregnancy process (history of pregnancy, gestational period), pathological history (presence of diseases), and information on oral care (visits to the dentist, having received health education, frequency of brushing and use of hygiene aids). 

The outcome variable was the presence of gingivitis, where a case was taken when clinical probing of the periodontium revealed a pocket depth ≤ 3 mm and bleeding ≥ 10%, based on the new classification of gingival health and biofilm-induced gingivitis.^23^ Six representative dental organs were taken as reference with the nomenclature of the International Dental Federation,^24^ these were 12,16,24,32,36 and 44. The periodontal examination was performed with an 11 mm coded probe (Hu-Friedy).

Four zones were considered: medial (lingual or palatal), distal, middle and mesial (buccal). Also, gingivitis status was established, which took the following ordinal values: probing depth ≤ 3 mm with <10% bleeding=healthy periodontium; probing depth ≤ 3 mm with ≥ 10% bleeding (two zones) in one or two teeth=localized gingivitis; and probing depth ≤ 3 mm with ≥ 10% bleeding (three or more zones) in three teeth or more=gingivitis generalized. 

To estimate O'Leary's simplified oral biofilm index, each pregnant woman was given a disclosing tablet to dissolve in the mouth and stain the dental surfaces with biofilm. ^(^^25^ The pigmented areas were recorded in the corresponding format. Six representative teeth were taken, which were the antagonists of those used for the gingival index (42,46,34,22,26 and 14) and were divided into four sectors (mesial, vestibular, distal and lingual or palatal). 

The number of pigmented faces was divided by the total number of total faces of the six teeth and multiplied by 100 to obtain the following nominal parameters: oral biofilm ≤ 29%=acceptable hygiene; and oral biofilm ≥ 30%=poor hygiene.

Other relevant operational variables included household income. An item on a rough estimate of income in Mexican pesos per month was applied. The values followed three ordinal categories, which were: income from $1000.00 to $7500.00 pesos=low; $7600.00 to 20,000.00 pesos=medium; and 21,000.00 or more pesos=high.

In order to avoid typo mistakes, data were double captured and validated with the EpiData V 3.1 program. ^(^^26^ The CIETmap program was used for statistical analysis of the data. ^(^^27^ Univariate analysis was performed to obtain simple frequencies of the study variables. 

The odds ratio and its 95% confidence interval of Miettinen were estimated as a measure of association between potential factors and the outcome variable. ^(^^28^ Through explanatory multivariate analysis, a model of factors associated with the presence of gingivitis was produced with the simultaneous analysis of the Mantel-Haenszel procedure. ^(^^29^ The initial saturated model included all the variables that had a significant association in the bivariate analysis, and adjusted for those considered by plausibility or consistency criteria.

The research protocol was approved by the Department of Teaching and Research of the Health Jurisdiction 07 of Acapulco, Guerrero. The filling out of the clinical record and the oral inspection were carried out in a dental cubicle, with the prior informed consent of the pregnant women, based on the Official Mexican Standard 004 of 2012 of the Ministry of Health. ^(^^30^ The research adhered to the ethical principles of the Helsinki Declaration of the World Medical Association. ^(^^31^ Once the pregnant woman had answered the questionnaire, the dental interns performed the oral inspection during the first hours of the morning. The clinical examination was non-invasive, without any health risk, and with the use of individual biosecurity measures. The oral health diagnosis was shared for the purpose of case control.

## RESULTS

Data were recorded for 92 pregnant women between 18 and 35 years of age, with a mean of 25.47 (SD 4.1). The 51% (47/92) were from urban areas and the rest from rural areas. Regarding the level of schooling, 36% (33/92) had basic education, 26% (24/92) had high school, 21% (19/92) had higher education and the rest had no education. Regarding marital status, 40% (37/92) are married, 25% (23/92) are single, 23% (21/92) are unmarried, 10% (9/92) are divorced and 2% (2/92) are widowed.

When classifying household income, the 55% (51/92) were low, 37% (34/92) medium and 8% (7/92) high. The 70% (64/92) of the pregnant women said that this was their first pregnancy and the rest had already had a pregnancy. Regarding the gestational period, 50% (46/92) were in the third trimester, 26% (24/92) and 24% (22/92) in the second and first trimester, respectively. The 24% (22/92) reported having a disease and the rest were apparently healthy. Of the diseases described, 59% (13/22) were hypertension, 32% (7/22) diabetes and 9% (2/22) both.

The 62% (57/92) of the pregnant women had not attended dental services during their pregnancy and the rest had. Only 22% (20/92) of the pregnant women had received health education. The frequency of brushing ranged from 1 to 3 times with a mean of 3.29 (SD 0.6); 47% (43/92) brushed two times per day, 41% (38/92) three times and 12% (11/92) one time. Regarding the use of oral hygiene aids, 71% (65/92) used them and 29% (27/92) did not use them. The 35% (23/65) use floss and oral rinse together, 23% (15/65) flosser, 22% (14/65) floss only and 20% (13/65) oral rinse only. Gingival disease was present in 60% (55/92) of the pregnant women, and the rest had healthy periodontium (Table 1). The 49% (45/92) of the pregnant women had poor dental hygiene, and the rest were acceptable. The percentages obtained ranged from 4% to 96%, with an overall total average of 38% (SD 0.21). 

**Table 1:** Parameters obtained for gingival disease among pregnant women.

Criteria*	Gingivitis Classification	Frequency n=92	%
Depth ≤ 3 mm with <10% bleeding	Healthy	37	40%
Depht ≤ 3 mm with ≥ 10% bleeding in 1-2 teeth	Localized	16	17%
Depht ≤ 3 mm with ≥ 10% bleeding in ≥ 3 teeth	Generalized	39	43%
Total		92	100%

* Parameters of the new classification of gingival health and biofilm-induced gingivitis.

Bivariate analysis identified five factors associated with the presence of gingivitis among pregnant women. The unadjusted association estimate and 95% confidence interval are shown in Table 2. The five relevant factors of the bivariate analysis, adjusted for the variables gestation period and O'Leary index, were included in the initial saturated model of the multivariate analysis. Only two factors were kept in the final model, which showed independence. The main associated strength was found in the variable not using oral hygiene aids (ORa=6.76; CI 95%=2.01-22.78) and the other was not attending dental services (ORa=3.74; CI 95%=1.44-9.73). Table 3 shows the adjusted estimate of the strength of association with its 95% confidence interval. The results exclude the counfounder effect and the X^2^ test for heterogeneity was greater than 0.05 for all associations in the final model, which excludes the existence of effect modification between the strata of the variables included.

**Table 2:** Bivariate analysis of factors associated with gingivitis among pregnant women.

Factor		Gingivitis		Healthy		ORna	CI 95%
		n=55	(%)	n=37	(%)		
Age	18-25 years^ref^	28	30%	17	19%	1.22	0.53 - 2.82
	26-35 years	27	29%	20	22%		
Location	Rural^ref^	30	33%	15	16%	1.76	0.76 - 4.10
	Urban	25	27%	22	24%		
Grade of studies	No education, elementary and high school^ref^	49	53%	24	26%	4.42	1.56 - 12.53*
	Superior	6	7%	13	14%		
Marital status	Single, divorced, widowed^ref^	21	23%	13	14%	1.14	0.48 - 2.73
	Married or common-law	34	37%	24	26%		
Household income	Low^ref^	51	55%	34	40%	1.12	0.23 - 5.39
	Medium- high	4	4%	3	1%		
First pregnancy	Yes^ref^	14	15%	14	15%	0.56	0.23 - 1.38
	No	41	45%	23	25%		
Gestation period	2° y 3° quarter^ref^	42	46%	28	30%	1.04	0.39 - 2.77
	1° quarter	13	14%	9	10%		
Diseases	Yes^ref^	12	13%	10	11%	0.75	0.29 - 1.99
	No	43	47%	27	29%		
Dental visit	No^ref^	42	46%	15	16%	4.74	1.96 - 11.46*
	Yes	13	14%	22	24%		
Health education	Did not receive^ref^	49	53%	23	25%	4.97	1.78 - 13.91*
	Did receive	6	7%	14	15%		
Toothbrushing	< 3^ref^	40	43%	14	16%	4.38	1.83 - 10.49*
	≥ 3	15	16%	23	25%		
Oral hygiene aids	Does not use^ref^	31	34%	34	37%	8.77	2.73 - 28.17*
	Use	24	26%	3	3%		
O´Leary index	≥ 30%^ref^	29	31%	16	18%	1.46	0.63 - 3.38
	≤ 29%	26	28%	21	23%		

ORna= unadjusted odds ratio

CI 95% = confidence interval of 95%

**Table 3:** Final model of the multivariate analysis of factors associated with gingivitis among pregnant women.

Factor	ORa	CI 95%	X^2^ het	p
Do not attend dental services	3.74	1.44 - 9.73	7.34	0.751
Do not use oral hygiene aids	6.76	2.01 - 22.78	9.52	0.749

ORa = adjusted odds ratio.

CI 95% = confidence interval of 95%.

X^2^ het = chi-square of heterogeneity to evaluate effect modification.

p= value of the X^2^ of heterogeneity.

## DISCUSSION

The prevalence of gingivitis in pregnant women was high. Two risk factors were found, not using oral hygiene aids, which has almost seven times the risk of developing gingivitis. The other is not attending dental appointments during pregnancy with four times the risk of developing the disease.

This study, being a cross-sectional design, has limitations with respect to clarifying temporality. Regarding dental visits, not visiting the dental service during pregnancy increases the risk of gingivitis, similar to the findings of Onigbinde *et al*., ^(^^10^ among Nigerian pregnant women. However, the question was directed to visits during the gestational process, not knowing if the pregnant women already had the disease or if it occurred during pregnancy. Systematic reviews establish that regular dental check-ups reduce gingivitis rates from 16% to 83%.^18^^,^^21^

Regarding the other factor, not using oral hygiene aids, it would reasonably be expected that this would precede the effect, since this habit is acquired from an early age. The use of auxiliaries enters the primary level of specific protection through health education. ^(^^21^ Pereda-Rojas *et al*., ^(^^20^ suggest that educational intervention aimed at health promotion in pregnant women increases knowledge to prevent the development of oral diseases.

The prevalence of gingivitis of 60% was similar to that reported by Corchuelo-Ojeda *et al*., ^(^^13^ among Colombian pregnant women. It was also similar to that reported by García-Morales *et al*., ^(^^17^ in a public hospital from Acapulco. The difference between our study and theirs is that they did not document associated factors. Other studies report low rates of periodontal disease (range 12% to 40%);^7^^,^^12^ and others high (range 80% to 90%).^9^^,^^11^^,^^14^^,^^15^

According to the parameters obtained based on the index of the new classification of biofilm-associated gingivitis, we described that generalized gingivitis was the most frequent. The morbidity of gingivitis reported in other studies differs from our study because of the epidemiological indexes used. Some authors categorized the progression of periodontal disease, with moderate being the most frequent; ^(^^9^^,^^14^ and mild stage. ^(^^11^^,^^16^

As for age, gingival disease was frequent in the 18 to 25 years age group, equal to another. ^(^^14^ One study recorded that mild bleeding based on the community periodontal index and presence of calculus occurs in pregnant women aged 20 to 24 years. ^(^^10^ Erchick *et al*., ^(^^7^ documented that gingivitis has a 3% increase in each year of age, in women with short stature and without a history of childbirth. On the other hand, Chen *et al*., ^(^^9^ concluded that the severity of gingivitis increases with age and with gingival bleeding during brushing. 

Gingivitis was higher during the second and third trimester of gestation, where this variable was included for consistency criteria, but no association was found. One documented that in the second trimester of pregnancy, there is a greater probability of periodontal disease settling. ^(^^10^ Other studies mention the first, ^(^^14^ and third trimester of gestation. ^(^^8^^,^^15^ Hormonal changes are involved in the appearance of gingivitis, therefore, clinical monitoring reduces conditions in pregnant women. ^(^^1^^,^^2^^,^^3^^,^^6^

Regarding the biofilm index, we found that 49% of the pregnant women had poor oral hygiene. The variable was included by biological plausibility criteria in the final model of the multivariate analysis; however, we did not find a significant association. Other studies agree that poor oral hygiene favors the development of gingivitis. ^(^^5^^,^^14^^,^^17^

The percentages of oral biofilm are similar to the presence of periodontal disease. ^(^^17^ The lack of dental visits during pregnancy increases the exacerbation of biofilm and gingivitis. ^(^^10^^,^^18^ In our study, 59% of pregnant women brush their teeth one or two times per day and 29% do not use hygiene aids, therefore, this increases the presence of the condition. It is important to eliminate irritating factors, since they increase the epidemiological characteristics of periodontal disease and influence the loss of gingival attachment. ^(^^7^^,^^15^

Other studies report socioeconomic, ^(^^5^^,^^7^^,^^12^ and racial^5^^,^^13^ determinants as factors associated with the presence of gingivitis during pregnancy. We asked about the level of schooling and place of origin, where most of the pregnant women were from rural areas and had no education or even higher education, however, no significant association was demonstrated.

Of concern was that 78% of the pregnant women had not received oral health education. Geisinger *et al*., ^(^^21^ mention that health education decreases oral biofilm and gingival inflammation from 50% to 40%, respectively. Therefore, health education is a strategy that reduces periodontal disorders in pregnant women. ^(^^19^^-^^21^

We accept that the major drawback of the study was obtaining a convenience sample, so the results are not representative of the population and cannot be extrapolated. It is not possible to compare the results with the population attending public services or private hospitals situated in the center of the city, since the population has different characteristics, being a private clinic located on the outskirts of the city. Although the sample is not representative of the population, initial trends have been identified that maintain the internal consistency of the study and will lay the foundations for following up a line of research in future studies.

## CONCLUSION

A prevalence of gingivitis of 60% was reported, similar to other studies. Regarding the risk factors identified, it is important to provide health education to encourage the use of oral hygiene aids. In addition, the dentist should emphasize to pregnant women the importance of clinical monitoring during the gestational period.
